# Crosstalk between Thyroid Hormone Receptor and Liver X Receptor in the Regulation of Selective Alzheimer’s Disease Indicator-1 Gene Expression

**DOI:** 10.1371/journal.pone.0054901

**Published:** 2013-01-24

**Authors:** Emi Ishida, Koshi Hashimoto, Shuichi Okada, Tetsurou Satoh, Masanobu Yamada, Masatomo Mori

**Affiliations:** Department of Medicine and Molecular Science, Gunma University Graduate School of Medicine, Maebashi, Gunma, Japan; University Claude Bernard Lyon 1, France

## Abstract

Selective Alzheimer’s disease (AD) indicator 1 (Seladin-1) has been identified as a gene down-regulated in the degenerated lesions of AD brain. Up-regulation of Seladin-1 reduces the accumulation of β-amyloid and neuronal death. Thyroid hormone (TH) exerts an important effect on the development and maintenance of central nervous systems. In the current study, we demonstrated that Seladin-1 gene and protein expression in the forebrain was increased in thyrotoxic mice compared with that of euthyroid mice. However, unexpectedly, no significant decrease in the gene and protein expression was observed in hypothyroid mice. Interestingly, an agonist of liver X receptor (LXR), TO901317 (TO) administration *in vivo* increased Seladin-1 gene and protein expression in the mouse forebrain only in a hypothyroid state and in the presence of mutant TR-β, suggesting that LXR-α would compensate for TR-β function to maintain Seladin-1 gene expression in hypothyroidism and resistance to TH. TH activated the mouse Seladin-1 gene promoter (−1936/+21 bp) and site 2 including canonical TH response element (TRE) half-site in the region between −159 and −154 bp is responsible for the positive regulation. RXR-α/TR-β heterodimerization was identified on site 2 by gel-shift assay, and chromatin immunoprecipitation assay revealed the recruitment of TR-β to site 2 and the recruitment was increased upon TH administration. On the other hand, LXR-α utilizes a distinct region from site 2 (−120 to −102 bp) to activate the mouse Seladin-1 gene promoter. Taking these findings together, we concluded that TH up-regulates Seladin-1 gene expression at the transcriptional level and LXR-α maintains the gene expression.

## Introduction

Alzheimer’s disease (AD) is one of the major causes of dementia and a serious concern to the human society [1], [2]. However, the pathogenesis of the disease has not yet been revealed.

Thyroid hormone (TH) is well known to play an important role in the development and maintenance of the central nervous system in mammals [3], [4]. TH exerts its biological function through thyroid hormone receptors (TRs). TRs are nuclear hormone receptors, to which triiodothyronine (T_3_) binds at a high-affinity order as a native ligand. TRs possess at least two isoforms, TR-α and -β (Nr1a1 and Nr1a2), and several isoforms exist as two or three subtypes, respectively (α1, α2, β1, β2, and β3) [5]. It is of note that only TR-α1, β1 and β2 have both a ligand binding and a DNA binding domain [6]. TR-α1 is widely expressed in tissues including heart, muscle, intestine, bone, and brain and plays a key role in regulating postnatal development and cardiac metabolism, whereas TR-β1 is also widely expressed in brain, cochlea, pituitary, kidney, lung, heart and at its highest level in the liver regulating multiple steps in hepatic metabolism as well as thyroid hormone levels [6]. TR-β2 expression is mainly in the pituitary, the hypothalamic TRH neurons, the developing inner ear and retina [7]. Thus, both TR-α and TR-β play an important role for the development and the maintenance of the central nervous system even though their expression patterns are spatiotemporally distinct [8]–[10]. Hypothyroidism sometimes leads to the development of dementia-like symptoms, especially in elderly patients [11], [12]. The TH receptor (TR) β-Δ337T knock-in (TRKI) mouse demonstrates severe cerebellar ataxia and cognitive dysfunction [13]. As such, although case reports and basic studies support the idea that TH is closely related to AD pathogenesis and could be beneficial to cure AD [14]–[16], large-scale clinical studies examining the relationship between thyroid function and AD have led to controversial conclusions [17]–[20].

Among many genes related to AD, we focused on selective AD indicator 1, Seladin-1 gene. Seladin-1 gene expression is down-regulated in the vulnerable region in the brain of AD patients [21]. Up-regulation of Seladin-1 in the neuron leads to the reduction of β-amyloid accumulation and apoptosis [21]–[23]. Seladin-1 gene codes 24-dehydrocholesterol reductase (DHCR24), which catalyzes the final step of cholesterol synthesis [24]. Cholesterol inside the neuron inhibits co-localization of β-amyloid precursor protein (APP) and β-site APP-cleaving enzyme (BACE), protecting β-amyloid accumulation [22], [25]. Thus, cholesterol in the neuron is responsible for maintenance of neural function [25]. Seladin-1 protects the neuron by increasing the intracellular cholesterol synthesis [21], [22], [26]. In a previous report, TH administration to some neuronal precursor cell lines induced Seladin-1 gene expression [27]. However, the molecular mechanism by which TH up-regulates Seladin-1 mRNA levels is yet to be elucidated.

Liver X receptors (LXRs) are nuclear oxysterol receptors and play pivotal roles in cholesterol metabolism [28], [29]. LXRs comprise two isoforms, LXR-α and -β. Both isoforms are expressed in the brain, although the latter is expressed at significantly higher levels [30]. An artificial agonist of LXR, TO901317 (TO), reduces β-amyloid accumulation and AD-specific pathological changes in the brain of an AD model mouse [31]–[33]. Since LXR response element (LXRE) is identified in the second intron in human Seladin-1 gene promoter, another group has suggested that Seladin-1 gene promoter may be a target of LXR [34]. However, the molecular mechanism by which LXRs regulate Seladin-1 gene expression has been yet to be elucidated.

TRs and LXRs are members of the nuclear receptor superfamily. Although LXRs and TRs belong to two distinct receptor subgroups with respect to ligand-binding affinity [35], the two receptor systems show similarity with respect to molecular mechanism, target genes, and physiological roles [36]. Since both TRs and LXRs play an important role in metabolic regulation, form heterodimers with retinoid X receptors (RXRs), and bind to direct repeat-4 (DR-4) with identical geometry and polarity [28], [36]–[38], crosstalk between the two receptors has been reported, especially on lipid metabolism-related genes [37]–[43].

In the current study, we demonstrate that TH increased Seladin-1 gene and protein expression in the mouse forebrain. However, no significant difference between euthyroid and hypothyroid mice was observed. Interestingly, TO administration *in vivo* increased Seladin-1 gene and protein expression in the mouse forebrain only in a hypothyroid state and in the presence of mutant TR-β. We also show that TH activates mouse Seladin-1 gene promoter and identify a novel positive thyroid hormone response element named site 2 including canonical thyroid hormone response element (TRE) half-site in the mouse promoter. Taking these findings together, we concluded that TH up-regulates Seladin-1 gene expression at the transcriptional level and that LXR-α would compensate for TR-β function to maintain Seladin-1 gene expression in hypothyroidism and resistance to TH (RTH). Thus, our data strongly suggests that TR and LXR crosstalk in Seladin-1 gene regulation.

## Materials and Methods

### Animals

8 to 12-week-old male C57/BL6 (B6) and TRKI mice (generously provided by Prof. Fredric E. Wondisford, Johns Hopkins medical institute, Baltimore) were employed for the study. All aspects of animal care were approved by the Institutional Animal Care and Use Committee of Gunma University Graduate School of Medicine (Maebashi, Gunma, Japan, Protocol number #10-059). Animals were maintained on a 12 h light/12 h dark schedule (lights on at 06∶00 h) and fed laboratory chow as indicated and given water ad libitum. Hypothyroidism was induced in the B6 mice by the inclusion of 0.1% methimazole (MMI) in the drinking water and 1% (w/w) propylthiouracil (PTU) in the chow for 21 days [37], [41]. To induce a thyrotoxic state, the B6 mice were injected daily with 10 µg per 20 g body weight of T_3_ for 7 days [37]. For TO treatment, 1 mg per 20 g body weight of TO901317 (#71810 Cayman, Ann Arbor, MI) was intraperitoneally administered daily for 7 days [31], [33] or vehicle (DMSO) was administered for Sham group. The number of mice receiving each treatment is indicated in the figures. Serum-free T_3_ and T_4_ levels were measured by SRL (Tokyo, Japan). We anatomically excised cerebellum, diencephalon, pituitary, hypothalamus, and medulla oblongata from the whole brain of mouse and obtained the rest as forebrain for the analysis as we reported previously [13].

### Plasmids

The mouse Seladin-1 gene promoter (−1936/+21 bp) plasmid, which contained the region from −1936 to +21 bp of the mouse Seladin-1 gene, was generated by genomic PCR using 5′-GTGTGGTACCTGGTGGCAGGAGAGAGCCCC-3′ as a sense primer and 5′-GTGTAGATCTGTAAGTCGGGCCCGCCGCC-3′ as an anti-sense primer. An *Asp718* or *BglII* restriction enzyme site was introduced into each primer sequence so that the PCR product could be subcloned between these sites in the pGL4-Luc vector (Promega, Madison, WI). The deletion mutants and a site 1 or site 2 point mutants were generated with PCR site-directed mutagenesis [37], [43], [44]. All human TR-β1 and its mutants, human TR-α1, RXR-α, murine LXR-α, and LXR-β cDNAs were placed into an SV40 expression construct, pSG5 [44]. All PCR-generated constructs were verified by sequencing the DNA.

### Transfections and Luciferase Assay

For the luciferase assay, we employed CV-1 cells. 10 µg of the reporter plasmid and 0.25 µg of human TR-β1 or TR-α1 or mouse LXR-α1 or LXR-β in pSG5 was transfected per plate of a 6-well format into CV-1 cells using the calcium-phosphate method. Sixteen hours after transfection, cultures were treated with Dulbecco’s modified Eagle’s medium (DMEM) containing 10% resin charcoal double-stripped fetal bovine serum for 8 hours in the absence or presence of 10^−8^ M T_3_ or 10^−6^ M TO. All transfections were equalized for the same total amount of expression vector using an empty vector as needed. We performed β-gal assays to confirm the transfection efficiency of the luciferase assay for each experiment at least once and found no significant difference in transfection efficiency among the plates. Data are presented as fold basal activation over vector (pSG5) in the absence of ligand stimulation ± SEM or as otherwise indicated. Luciferase activity is expressed as arbitrary light units per µg of cellular protein. All transfection experiments were repeated at least twice with triplicate determinations.

### Western Blotting

For analysis of the protein expression of Seladin-1 (DHCR24), 30 µg of whole cell extract from mouse forebrain was subjected to SDS-PAGE. Western blotting was performed using a rabbit anti-DHCR24 polyclonal antibody (#ab40490, Abcam, Cambridge, UK) to detect Sealdin-1 protein levels and anti-glyceraldehyde-3-phosphate dehydrogenase (GAPDH) (#ab8245, Abcam, Cambridge, UK) as a control. The Seladin-1 detects a specific band at 55 kDa in tissues and cells. The bands were quantitatively measured using Adobe Photoshop 7.0 (Adobe Systems Corp., San Jose, CA) and ImageJ (Rasband, W.S., ImageJ, US National Institutes of Health, Bethesda, Maryland, USA, http://imagej.nih.gov/ij/, 1997–2011). All Western blotting experiments were repeated at least three times with similar results. Seladin-1 protein levels are normalized by GAPDH. Data are presented as fold basal euthyroid (E) levels ± SEM or as otherwise indicated.

### RNA Preparation and Real-time Quantitative PCR

Total RNA was extracted from mouse forebrain using ISOGEN (Nippon Gene, Tokyo, Japan). Real-time quantitative PCR assays were performed using an Applied Biosystems 7500 sequence detector with the standard 40 cycles. Briefly, 1 µg of total RNA was reverse-transcribed with random hexamers using the Taqman^TM^ Reverse Transcription Reagent kit (Applied Biosystems, Carlsbad, CA) according to the manufacturer’s protocol. Mouse Seladin-1 mRNA expression was analyzed using Taqman^TM^ probes (Mm00519071_m1, Applied Biosystems, Carlsbad, CA). We set up a standard curve for each real-time PCR and confirmed that all PCR products were on the standard curve as we previously described [43]. The PCR results were normalized to mouse cyclophilin A expression using a Taqman^TM^ probe (Mm02342430_gl, Applied Biosystems, Carlsbad, CA). The number of samples is indicated in the figure legends.

### Gel-shift Assays

Electrophoretic mobility-shift assays (EMSAs, gel-shift assays) were performed as described previously [45]. Mouse LXR-α, human TR-β1, and human RXR-α recombinant proteins were synthesized from constructs in the pSG5 expression vector, using the TNT T7 Quick Coupled Transcription/ Translation System (Promega, Madison, WI). Binding reactions contained 20 mM HEPES (pH 7.6), 50 mM KCl, 12% glycerol, 1 mM dithiothreitol (DTT), 1 µg of poly (dI-dC)(dI-dC), and 6 µl of each of the synthesized nuclear receptors or unprogrammed reticulocyte lysates. Double-stranded oligonucleotides (DR-4 for rat cholesterol 7α-hydroxylase (CYP7A1): 5′- TGTTTGCTTTGGTCACTCAAGTTCAA-3′, Site 1 probe ; 5′- CTCCCACCCTGGGGAGGTTACCCAATGCACAACT-3′, Site 2 probe ; 5′- AGCGCCCCCAGCCCAGGTCAGCCCCTCCTCGCAC-3′) were labeled with [α-^32^P] deoxy-CTP by a fill-in reaction using a Klenow fragment of DNA polymerase. Binding reactions were performed at room temperature for 30 min. For competition experiments, a 20- or 40-fold molar excess of cold oligonucleotides was included. For supershift experiments, 3 µl of rabbit anti-TR-β1 polyclonal antibody (06-539, Upstate, Lake Placid, NY) or mouse anti-LXR-α monoclonal antibody (PP-PPZ0412-00, Perseus proteomics, Tokyo, Japan) was added and the mixture was incubated for an additional 30 min at room temperature. The protein-DNA complexes were resolved on a 4% polyacrylamide gel in 0.5X TBE (45 mM Tris-base, 1 mM EDTA). All gel-shift assays were repeated at least three times with similar results and a representative result is shown.

### Small Interfering (si) RNA against LXRs

We employed Dharmacon’s siRNA against human LXR-α (L-003413-00-0005, ON-TARGET plus SMART pool, human NR1H3) and LXR-β (L-003412-00-0005, ON-TARGET plus SMART pool, human NR1H2) (Thermo Fisher Scientific Inc., Waltham, MA). The target sequences of siRNA for human LXR-α are as follows: 5′-GAGUUUGCCUUGCUCAUUG-3′; 5′-CGACUGAUGUUCCCACGGA-3′; 5′-GAACAACUGGGCAUGAUCG-3′; 5′-CCUCAAGGAUUUCAGUUAU-3′, and the target sequences for LXR-β were as follows: 5′-AACAGCGGCUCAAGAACUA-3′; 5′-CUAAGCAAGUGCCUGGUUU-3′; 5′-GAAGAAGAUUCGGAAACAG-3′; 5′-CAACCACGAGACAGAGUGU-3′. SiRNA for the negative control (ON-TARGET plus Non-targeting pool, D-001810-10-20, Thermo Fisher Scientific, Inc., Waltham, MA) was employed. SiRNAs were transfected into HTB185 cells, derived from human medulloblastoma using the lipofection method (Lipofectamine RNAiMAX, Invitrogen). Briefly, in the 6-well format, 50 pmol of siRNAs against each LXR (α or β) or 100 pmol of non-targeting siRNA per well were transfected into the HTB185 cells. Sixteen hours after transfection, cultures were treated with DMEM containing 10% resin charcoal double-stripped fetal bovine serum for 8 hours in the absence or presence of 10^−6^ M TO. After incubation, total RNA was extracted from the cells and real-time quantitative PCR assays were performed as described above. Human Seladin-1, LXR-α and LXR-β mRNA expression levels were analyzed using Taqman^TM^ probes (Hs00207388_m1, Hs00172885_m1 and Hs00173195_m1, respectively. Applied Biosystems, Carlsbad, CA).

### Chromatin-immunoprecipitation (ChIP) Assay

ChIP assays were performed using a kit (ChIP-IT^TM^ Express) from Active Motif (Carlsbad, CA) in accordance with the manufacturer’s protocol with some modifications. Briefly, in prior to ChIP assays, the mouse Seladin-1 gene promoter (−1113/+21 bp) in pGL4 was transfected into the HTB185 cells with Lipofectamine 2000 (Invitrogen, Carlsbad, CA). The transfected HTB185 cells were incubated in medium containing 10% resin-charcoal double-stripped fetal bovine serum with or without 10^−8^ M T_3_. After incubation, formaldehyde (37%) was directly added to the culture at a final concentration of 1% and the cells were incubated for 15 min at 37°C to crosslink protein to DNA. The cells were pelleted and resuspended in 600 µl of lysis buffer supplemented with 3 µl of Protease Inhibitor Cocktail (PIC) and 0.5 mM PMSF and incubated on ice for 10 minutes. The resuspended cells were passed through a 27G syringe 10 times to aid nucleus release. The nucleus pellet was resuspended in 210 µl of Shearing Buffer (supplemented with 1.05 µl of PIC) and the samples were placed on ice. The nucleus lysate was sonicated 4 times with 10-sec pulses using a sonicator set at 50% of maximum power to reduce DNA length to between 200 and 1000 bp. Sheared chromatin solution (50 µl) was used for each ChIP assay with 2 µg of a mouse anti-TR-β monoclonal antibody (PP-H3825A-00, Perseus proteomics, Tokyo, Japan), mouse anti-LXR-α monoclonal antibody (PP-K8607-00, Perseus proteomics, Tokyo, Japan) and rabbit anti-RXR-α polyclonal antibody (sc-774, Santa Cruz Biotechnology, CA). As a negative control, normal rabbit IgG (#2729, Cell Signaling Technology, Denver, CO) was used. For Ct value-based evaluation of ChIP results, quantitative PCR was performed with power SYBR Green (#4367659, Applied Biosystems) in Applied Biosystems 7500 sequence detector by using 1 µl of immunoprecipitate or input according to the manufacturer’s specified parameters. The primers used for the region between −226 bp and +21 bp for quantitative PCR were as follows: forward 5′-CTCCAGAGCGAGAGCCCTAA-3′ and reverse 5′-GTAAGTCGGGCCCGCCGCCT-3′. The predicted PCR product was 247 bp long. The values were corrected using the input values and presented as %input ± SEM. All ChIP assays were repeated at least twice with triplicate determinations.

### Statistical Analyses

Statistical analysis was performed using ANOVA and parametric Welch’s *t*-test or nonparametric Mann-Whitney test [46], [47] where appropriate to assess statistical differences between means with Prism 5 (GraphPad Software, La Jolla, CA) and JMP (SAS Institute Inc., Cary, NC). Values are expressed as the mean ± standard error of the mean (SEM).

## Results

### T_3_ Up-regulates Seladin-1 Gene Expression in the Mouse Forebrain

**Figure 1:**
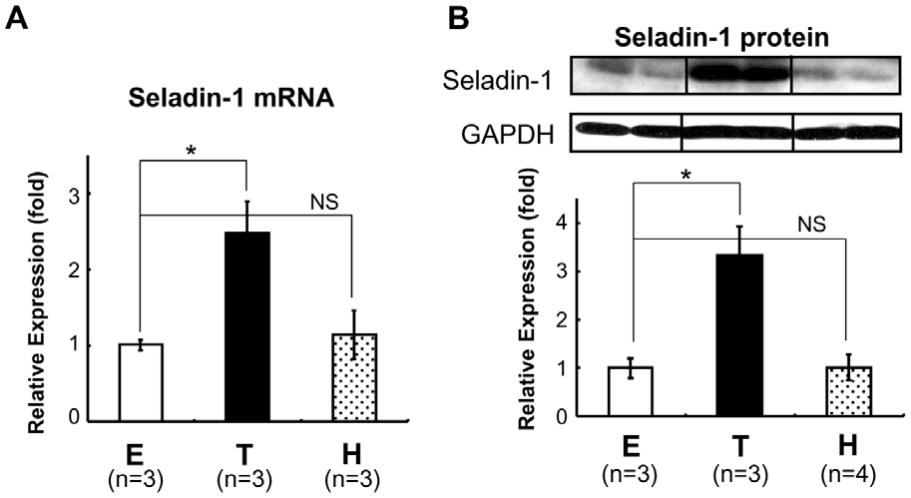
TH induced Seladin-1 mRNA and protein expression in the mouse forebrain. C57/B6 mice (8- to 12-week-old males) were rendered hypothyroid (H) with MMI and PTU diet or treated with T_3_ (T). E: euthyroid basal status (control). The number of mice involved in each treatment is indicated in the figures. Forebrain total RNA was isolated and one microgram of total RNA was subjected to reverse transcription (RT). RT real-time PCR analysis for Seladin-1 was performed using mouse forebrain cDNA (A). Relative values (mean ± S.E.) normalized by cyclophilin A mRNA levels compared with E are shown as relative expression (fold). Western blot analysis using mouse forebrain whole cell extract was performed (B). Representative Western blots for Seladin-1 are shown in the upper panel. Relative optical densities (O.D.) (mean ± S.E.) normalized by GAPDH levels compared with “E” are shown as relative expression (fold). An *asterisk* indicates that the difference between the denoted pairs is significant at a confidence level of *p<*0.05(*). NS: not significant.

To examine whether TH regulates mouse Seladin-1 gene expression, we performed real-time RT-PCR using mouse forebrain steady-state total RNA. For this purpose, we first rendered the B6 mice in a hypothyroid state (H) with an MMI/PTU diet, or made them thyrotoxic (T) with T_3_ administration intraperitoneally (Figure 1A, 1B). TH levels (free T_4_ and free T_3_) are shown in Table 1. As shown in Figure 1A, when T_3_ was administered to 8- to 12-week-old B6 mice intraperitoneally, Seladin-1 gene expression levels in the forebrain were increased about 2.5-fold. Unexpectedly, mice in a hypothyroid state did not show significant down-regulation of Seladin-1 gene compared to the control. We obtained a similar result for protein expression levels (Figure 1B).

**Table 1:** Free T_4_ and free T_3_ levels in each thyroid state.

	Free T_4_ (ng/dL)	Free T_3_ (pg/mL)
E (Euthyroid)	1.23±0.03	3.42±0.12
T (Thyrotoxic)	0.28±0.07**	66.7±8.3*
H (Hypothyroid)	0.14±0.04**	1.45±0.13***

Results are expressed as mean ± SEM (n = 3).

An *asterisk* indicates that the difference compared with “E” is significant at a confidence level of *p*<0.05 (*), *p*<0.01 (**), *p*<0.001 (***), by Welch’s *t*-testing.

### TO Up-regulates Seladin-1 Gene Expression in the Mouse Forebrain Under Hypothyroid Status and in the Forebrain of TRKI Mice

**Figure 2:**
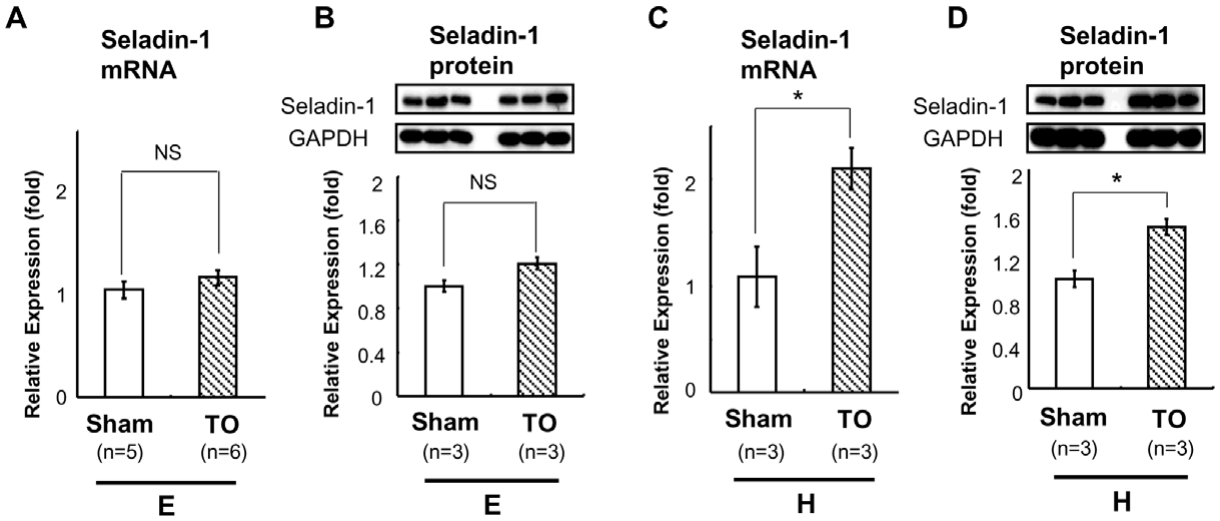
TO induced Seladin-1 mRNA and protein expression in the mouse forebrain in hypothyroid status. TO was administered to mice in euthyroid (E) or hypothyroid (H) status. The number of mice employed is indicated in the figures. Reverse transcription (RT) real-time PCR analysis for Seladin-1 was performed using mouse forebrain cDNA (A, C). Relative values (mean ± S.E.) normalized by cyclophilin A mRNA levels compared with Sham are shown as relative expression (fold). Western blot analysis using mouse forebrain whole cell extract was performed (B, D). Representative Western blots for Seladin-1 are shown in the upper panel (B, D). Relative values (mean ± S.E.) normalized by GAPDH protein levels compared with Sham are shown as relative expression (fold). An *asterisk* indicates that the difference between the denoted pairs is significant at a confidence level of *p<*0.05(*). NS: not significant.

**Figure 3:**
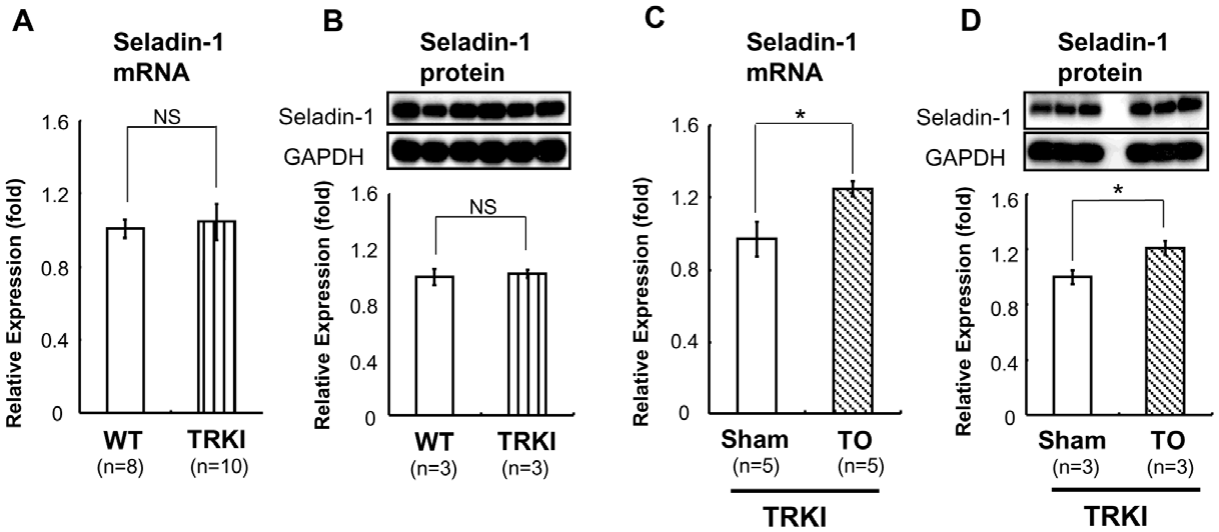
TO induced Seladin-1 mRNA and protein expression in the forebrain in TRKI mice. RT real-time PCR analysis for Seladin-1 was performed using mouse forebrain cDNA (A, C). Relative values (mean ± S.E.) normalized by cyclophilin A mRNA levels compared with Sham are shown as relative expression (fold). Western blot analysis using mouse forebrain whole cell extract was performed (B, D). Representative Western blots for Seladin-1 are shown in the upper panel (B, D). The number of mice employed is indicated in the figures. Relative values (mean ± S.E.) normalized by GAPDH protein levels compared with Sham are shown as relative expression (fold). An *asterisk* indicates that the difference between the denoted pairs is significant at a confidence level of *p<*0.05(*). NS: not significant.

Based on the data that hypothyroid treatment did not reduce Seladin-1 gene expression, from a perspective of TR-LXR crosstalk as previously reported [5], [37], [48], we speculated that LXR would compensate to maintain the gene expression under hypothyroid status. Unexpectedly, as shown in Figure 2A, TO did not change Seladin-1 gene expression levels in the forebrain of B6 mice in the basal status. However, in a hypothyroid state, TO increased Seladin-1 gene expression levels in the forebrain (by 2.1 fold, Figure 2C). We obtained a similar result for protein expression levels (Figure 2B, 2D). As previously reported [41], free T_4_ levels of TRKI mice (homozygous mice), which demonstrates severe thyroid dysfunction were extremely high (over 7.77 ng/dl) because of RTH (data not shown). However, Seladin-1 mRNA and protein levels in the forebrain of TRKI mice were comparable to those of wild-type (WT) mice (Figure 3A, 3B). On the other hand, as shown in Figure 3C, administration of TO significantly increased Seladin-1 gene expression in the forebrain of TRKI mice compared with that of Sham group about 1.2-fold. We obtained a similar result for protein expression levels (Figure 3D). These lines of evidence supported our hypothesis; LXRs compensate to maintain Seladin-1 gene expression in hypothyroidism and/or in TR-β dysfunction.

### T_3_ Activates Mouse Seladin-1 Gene Promoter via Novel Positive Thyroid Hormone Response Element (TRE), Site 2

**Figure 4:**
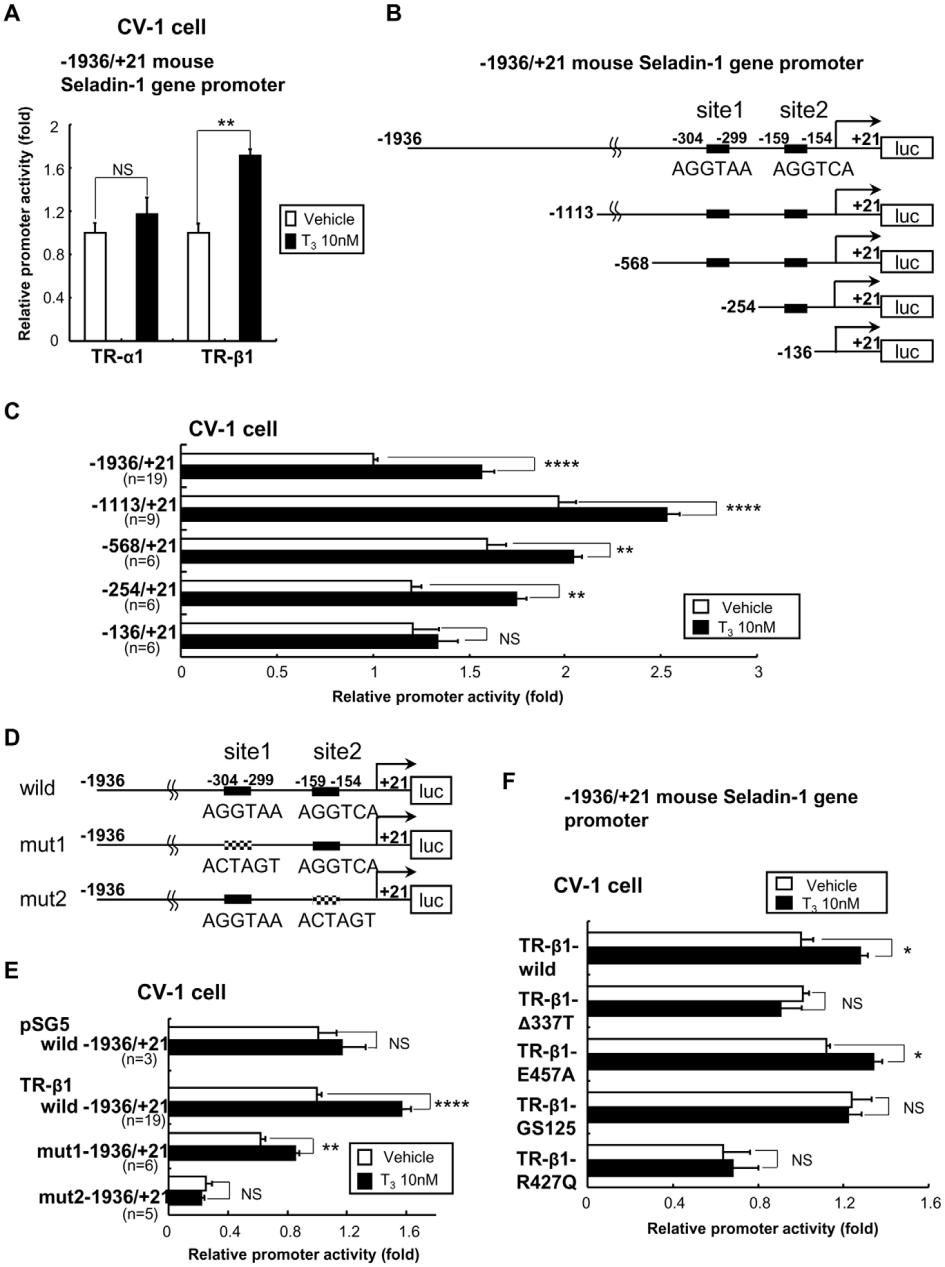
T_3_ induced the mouse Seladin-1 gene promoter activity via TR-β. A) T_3_ induced the mouse Seladin-1 gene promoter (−1936/+21 bp) coupled to the luciferase reporter construct (pGL4) in CV-1 cells in the presence of TR-β, but not TR-α. Vehicle for T_3_ was distilled water buffered with 1 mM HEPES (pH 7.5). Relative luciferase activities (mean ± S.E., n = 6) compared to the light units with the −1936/+21 bp construct in the absence of T_3_ (arbitrary light units divided by cellular protein and by β-gal activity) are shown as relative promoter activity (fold). B) A schematic representation of the deletion mutants of the mouse Seladin-1 gene promoter. The closed boxes indicate consensus TRE half-sites (site 1 and 2). C) The −136/+21 bp construct was not activated by T_3._ Relative luciferase activities (mean ± S.E.) compared to the light units with the −1936/+21 bp construct in the absence of T_3_ (arbitrary light units divided by cellular protein and by β-gal activity) are shown as relative promoter activity (fold). The number of samples is indicated in the figure. D) Hatched boxes indicate mutation in the TRE half-sites. E) Mutation in site 2 abolished the activation of the mouse Seladin-1 gene promoter by T_3_. Relative luciferase activities (mean ± S.E.) compared to the light units with the −1936/+21 bp construct and expression vector (pSG5) in the absence of T_3_ (arbitrary light units divided by cellular protein and by β-gal activity) are shown as relative promoter activity (fold). The number of samples is indicated in the figure. F) The mouse Seladin-1 gene promoter (−1936/+21 bp) reporter was co-transfected into CV-1 cells with several types of TR-β1 mutant. Relative luciferase activities (mean ± S.E., n = 3) compared with the light units with wild-type TR-β1 in the absence of T_3_ (arbitrary light units divided by cellular protein and by β-gal activity) are shown as relative promoter activity (fold). An *asterisk* indicates that the difference between the denoted pairs is significant at a confidence level of *p*<0.05(*), *p*<0.01(**), and *p*<0.0001(****). NS: not significant.

We subcloned mouse Seladin-1 gene promoter from −1936 to +21 base pair (bp) s and inserted it into luciferase reporter vector, pGL4 (Promega). We employed this reporter construct and performed luciferase assays with CV-1 cells, which are known to be deficient for endogenous TRs [45]. When we co-transfected the gene promoter (−1936/+21 bp) with TR-β1 into CV-1 cells, T_3_ significantly activated the promoter activity, however the up-regulation was not observed with co-transfection of TR-α1 (Figure 4A). These data indicated that TH activates mouse Seladin-1 gene promoter via TR-β. Next, we prepared a series of deletion constructs of the mouse Seladin-1 gene promoter (Figure 4B), which were subjected to transfection into CV-1 cells with these reporters together with TR-β1. As shown in Figure 4C, the −136/+21 construct was not activated by T_3_. We identified two TRE half-sites, one is located from −304 to −299 bp and the other one is located from −159 to −154 bp in the mouse Seladin-1 promoter and hypothesized that these TRE half site regions could be a novel positive TRE. We referred to the upstream and downstream region as site 1 and 2, respectively. We introduced mutation into the TRE half-site in site 1 and 2 in the context of the −1936/+21 reporter (Figure 4D, upper panel). As shown in Figure 4E, lower panel, the positive regulation by T_3_ deteriorated in the mutt 2 reporter, indicating that the TRE half-site in site 2 is functionally important.

### TR-β1 DNA Binding is Essential for Positive Regulation of Mouse Seladin-1 Gene Promoter

We co-transfected several types of mutant TR-β1 and the −1936/+21 reporter into CV-1 cells. As shown in Figure 4F, GS125, a DNA binding mutant [49], Δ337T, a ligand binding mutant [50], [51], and R427Q [51], [52], a heterodimerization mutant with RXR-α, did not activate the promoter, whereas E457A, a co-activator binding mutant [53], positively regulated mouse Seladin-1 gene promoter activity as wild-type TR-β1. These data suggest that ligand binding, TR-β1 DNA binding, and heterodimerization with RXR-α are required for the positive regulation.

### RXR-α/TR-β1 Heterodimer Binds to Site 2

**Figure 5:**
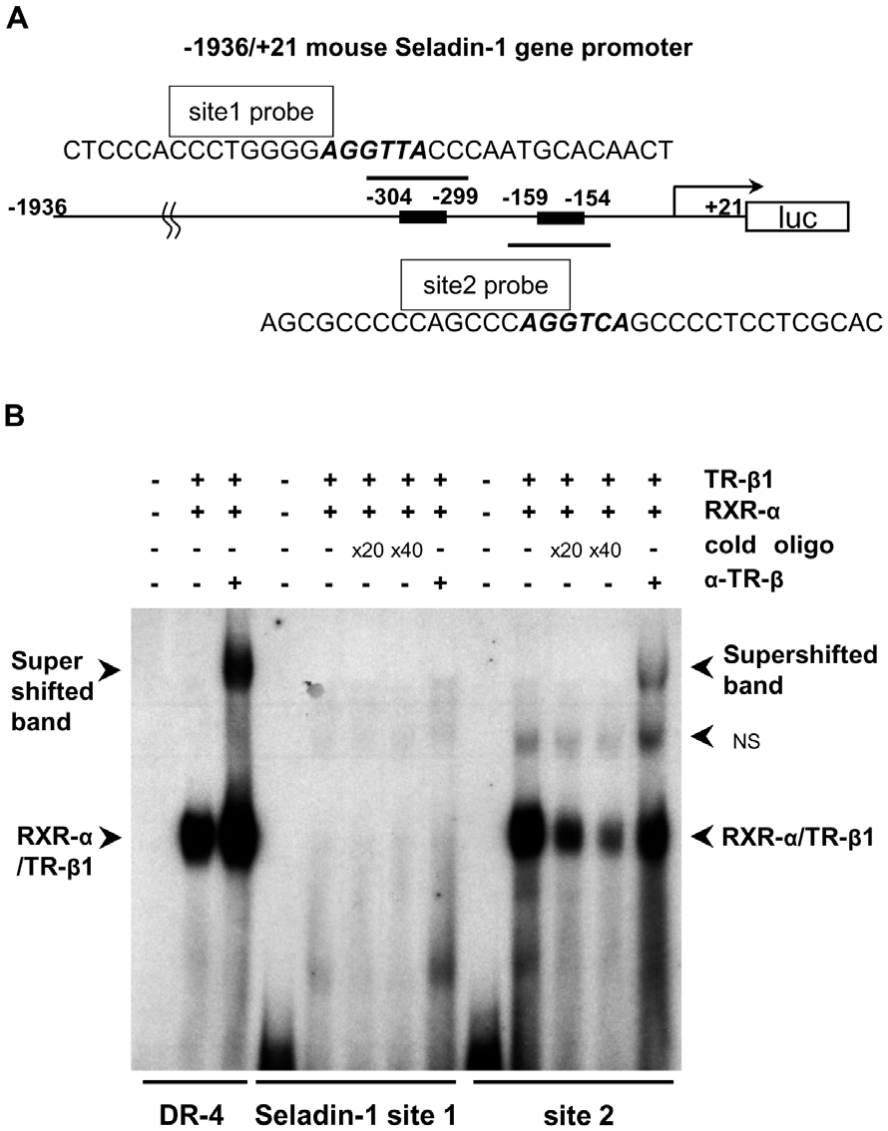
RXR-α/TR-β1 binds to site 2. Oligonucleotide sequences for radiolabelled probes are indicated in Figure 5A. Flanking regions of two TRE half sites were indicated as site 1 and 2 probes. The consensus TRE half sites were highlighted in italics. Six microliters (indicated as ‘+’) of *in vitro*-translated human TR-β1 and human RXR-α were incubated with ^32^P-radiolabeled DNA probes. For competition experiments, 20- or 40-fold molar excess of cold oligonucleotides was included as indicated. NS: non specific band. α-TR-β: rabbit anti-TR-β antibody. DR-4: Direct repeat-4.

Since we found that site 2 is functionally important for the positive regulation of the mouse Seladin-1 gene promoter based on the reporter assay data (Figure 4E), we hypothesized that TR-β1 could bind to site 2 in the mouse Seladin-1 gene promoter. To investigate this, we employed oligonucleotides for site 1, encompassing −318 to −285 bp and for site 2, from −173 to −140 bp in the mouse Seladin-1 gene promoter, including the TRE consensus half-site motif. We performed electrophoretic mobility-shift assays (EMSAs) with the radiolabeled site 1 and 2 probes (Figure 5A). As shown in Figure 5B, it was revealed that RXR-α/TR-β1 heterodimer bound to site 2 but not to site 1. Anti-TR-β antibody shifted the heterodimer band, indicating that the binding was TR-β-specific. In addition, either RXR-α/TR-α1 heterodimer or TR-α1 homodyne did not bind to site 2 (data not shown).

### TO Increased Seladin-1 Gene Expression in HTB185 Cells through Intrinsic LXRs

**Figure 6:**
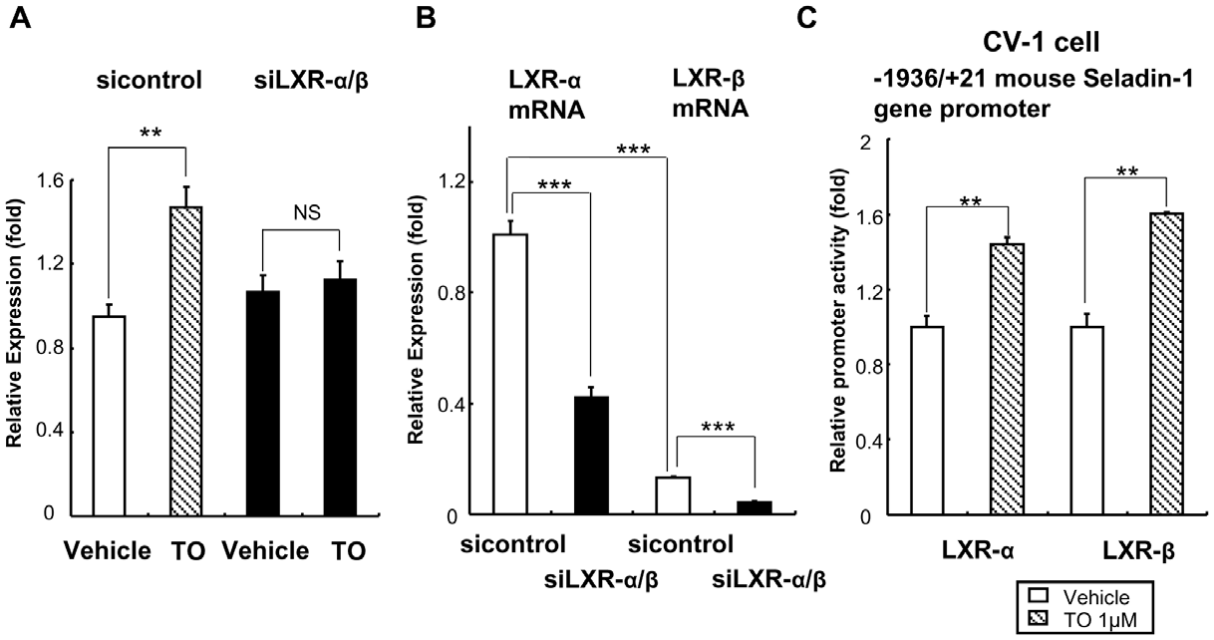
The up-regulation of Seladin-1 gene expression by TO requires LXRs in HTB185 cells. A) TO failed to induce the Seladin-1 gene expression in LXR-knockdown HTB185 cells. Vehicle for TO was DMSO. Relative values (mean ± S.E., n = 5) normalized by cyclophilin A mRNA levels compared with the expression levels of Seladin-1 mRNA with non-targeting siRNA (sicontrol) are shown as relative expression (fold). B) SiRNAs against LXR-α and LXR-β significantly reduced each gene expression level in HTB185 cells. Relative values (mean ± S.E., n = 6) normalized by cyclophilin A mRNA levels compared with the expression levels of LXR-α mRNA with sicontrol are shown as relative expression (fold). C) TO induced the mouse Seladin-1 gene promoter (−1936/+21 bp) in CV-1 cells in the presence of LXR-α or LXR-β. Relative luciferase activities (mean ± S.E., n = 3) compared to the light units with the −1936/+21 bp construct in the absence of TO (arbitrary light units divided by cellular protein and by β-gal activity) are shown as relative promoter activity (fold). An *asterisk* indicates that the difference between the denoted pairs is significant at a confidence level of *p*<0.05(*), *p*<0.01(**) and *p*<0.001(***). NS: not significant.

On the basis of the data that TO increased mouse Seladin-1 gene expression in the forebrain in hypothyroid state and in TRKI mice, we examined whether TO potentiate to increase Seladin-1 gene expression in HTB185 cells and requires intrinsic LXRs. As shown in Figure 6A, TO significantly increased human Seladin-1 gene expression about 1.5 fold (left graph). In addition, induction of siRNA against LXR-α/β into HTB185 cells revealed that TO did not increase the gene expression in LXRs knockdown-HTB185 cells (Figure 6A, right graph). These data indicate that TO increases Seladin-1 gene expression in HTB185 cells through intrinsic LXRs. We confirmed LXR-α and -β gene expression levels in the HTB 185 cells, which siRNA against LXR-α/β was induced (Figure 6B). It revealed that both LXR isoform gene expression levels were significantly reduced in the LXRs knockdown cells. In addition, TO induced the mouse Seladin-1 gene promoter (−1936/+21 bp) in CV-1 cells in the presence of LXR-α or LXR-β indicating that both isoforms potentiate to activate the gene promoter upon TO administration (Figure 6C). However, as shown in Figure 6B, since the gene expression of LXR-α was robust compared to that of LXR-β, we concluded that LXR-α is the dominant isoform in HTB185 cells. Thus, we employed LXR-α for further analysis of the up-regulation of mouse Seladin-1 gene promoter by TO.

### TO Activates Mouse Seladin-1 Gene Promoter, However LXR-α or RXR-α/LXR-α Heterodimer did not Bind to Site 2

**Figure 7:**
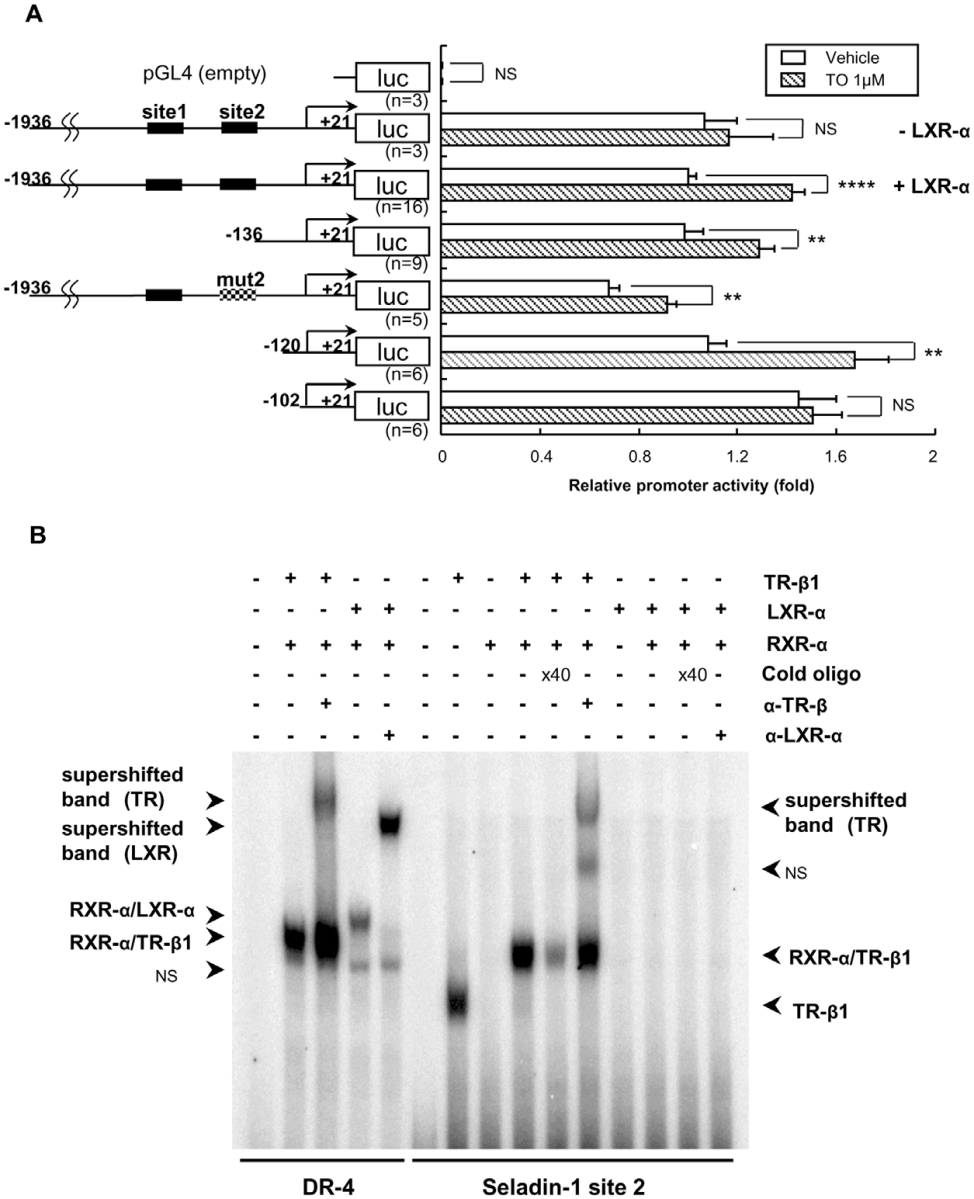
TO induced the mouse Seladin-1 gene promoter activity but LXR-α did not bind to site 2. A) A schematic representation of the deletion mutants and site 2 mutant (mut 2) of the mouse Seladin-1 gene promoter. The closed boxes indicate site 1 and 2. The hatched box indicates mutation in the TRE half-site. TO induced the mouse Seladin-1 gene promoter (−1936/+21 bp) coupled to the luciferase reporter construct (pGL4) in CV-1 cells in the presence of LXR-α. Vehicle for TO was DMSO. Relative luciferase activities (mean ± S.E.) compared with the light units of the wild mouse Seladin-1 promoter (−1936/+21 bp) reporter with LXR-α in the absence of TO (arbitrary light units divided by cellular protein and by β-gal activity) are shown as relative promoter activity (fold). The number of samples is indicated in the figure. An *asterisk* indicates that the difference between the denoted pairs is significant at a confidence level of *p*<0.01(**) and *p*<0.0001(****). NS: not significant. B) LXR-α or RXR-α/LXR-α does not bind to site 2. Six microliters (indicated as ‘+’) of *in vitro*-translated human TR-β1 and/or human RXR-α and/or mouse LXR-α protein were incubated with ^32^P-radiolabeled DNA probes. For competition experiments, 40-fold molar excess of cold oligonucleotides was included as indicated. NS: Non-specific band. α-TR-β: rabbit anti-TR-β antibody. α-LXR-α: mouse anti-LXR-α antibody. DR-4: Direct repeat-4.

As shown in Figure 7A, TO activates mouse Seladin-1 gene promoter (−1936 to +21 bp) in CV-1 cells upon co-transfection with LXR-α, which is a dominant isoform of LXRs in HTB185 cells. An analysis of deletion constructs of the gene promoter revealed that the −102/+21 construct was not activated by TO indicating that the region from −120 to −102 bp but not site 2 is responsible for the positive regulation (Figure 7A). Moreover, EMSA demonstrated that LXR-α or RXR-α/LXR-α heterodimer did not bind to site 2 (Figure 7B). We performed EMSA employing several probes with different sequence including the region from −120 to −102 bp; however, LXR-α or RXR-α/LXR-α heterodimer did not bind to any probes (data not shown).

### TR-β is Recruited to the Site 2 Region in the Mouse Seladin-1 Gene Promoter in a T_3_-dependent Manner

**Figure 8:**
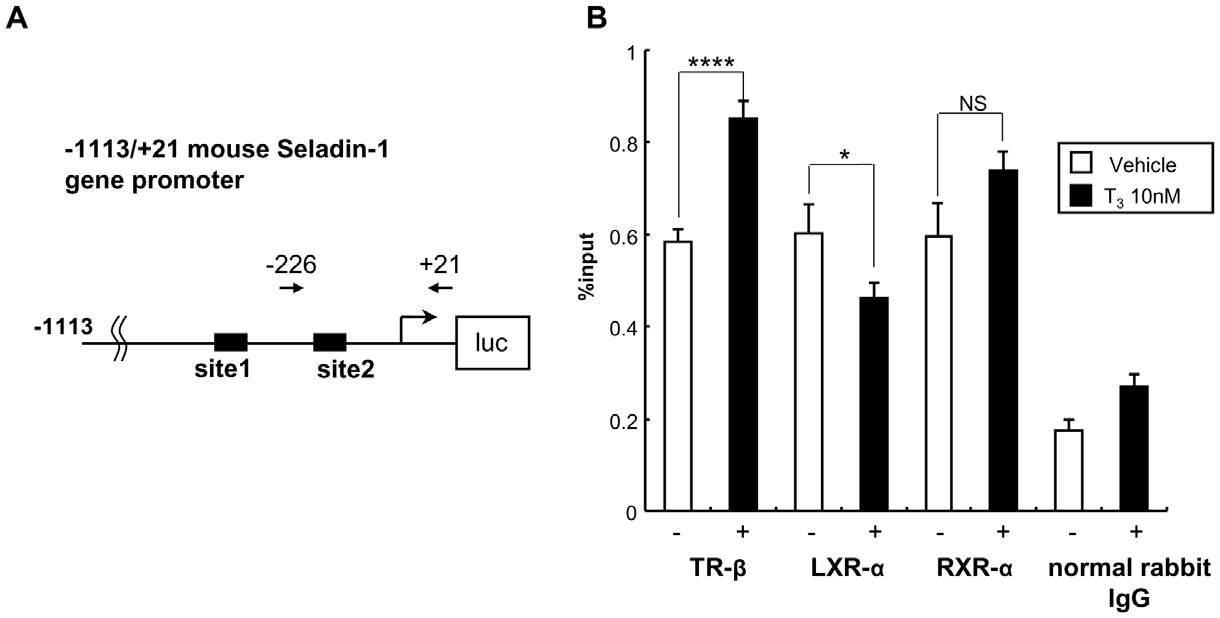
TR-β is recruited to the site 2 region in the mouse Seladin-1 gene promoter in a T_3_-dependent manner. The location of the PCR primers is indicated in an image above the data as arrows. The closed boxes indicate site 1 and 2 (A). Real-time PCR was performed to determine the relative value of the enrichment of each nuclear receptor. The value is shown as % of input (mean ± S.E., n = 8) (B). TR-β, RXR-α and LXR-α are recruited to the region from −226 bp to +21 bp. The recruitment of TR-β and RXR-α is increased upon T_3_ administration. The normal rabbit IgG was used as a negative control. An *asterisk* indicates that the difference between the denoted pairs is significant at a confidence level of *p*<0.05(*) and *p*<0.0001(****). NS: not significant.

To elucidate interaction between TR-β and the mouse Seladin-1 gene promoter, we performed chromatin immunoprecipitation (ChIP) assays using a set of primers amplifying the −226 to +21 bp region, which includes site 2, in the mouse Seladin-1 gene promoter followed by quantitative PCR (Figure 8A). We introduced the mouse Seladin-1 gene promoter (−1113 to +21 bp) in HTB185 cells by transient transfection. As shown in Figure 8B, in HTB185 cells, the recruitment of intrinsic TR-β to the −226 to +21 bp region significantly increased upon T_3_ administration. Since the set of PCR primer covers the region from −120 to −102 bp, with which LXR-α should interact, LXR-α recruitment to the region was observed in the absence of TH, but TH significantly reduced the recruitment.

## Discussion

In the current study, we demonstrated that mouse Seladin-1 mRNA and protein levels in forebrain were up-regulated by TH administration *in vivo*. We have obtained some controversial results from the *in vivo* study. While Seladin-1 gene expression is induced in a thyrotoxic state, under hypothyroid treatment, the gene and protein expression levels were not significantly reduced compared to those in a euthyroid state (Figure 1). Moreover, no significant differences in the gene expression between wild-type and TRKI mice, which was expected to recapitulate hypothyroid state in the forebrain, were found (Figure 3A and 3B). This *in vivo* data indicated a compensation to maintain the gene expression should exist. First, we tried to elucidate the molecular mechanism by which TH up-regulates the gene expression and we demonstrated that the mouse Seladin-1 gene promoter was activated by T_3_ in CV-1 cells. Benvenuti et al. reported that TH up-regulates the Seladin-1 gene expression in *in vitro* model of human neuronal precursor cell lines [27]. They conformed that TR-α1, TR-β1 and TR-β2 genes were expressed in these neuronal precursor cell lines, however it was unclear whether the up-regulation of the Seladin-1 gene expression by T_3_ was isoform specific. It is well known that both TR-α and TR-β are important for the development and maintenance of central nervous system in mammals and other species [7]–[10], [54]. Thus, we examined the activation of the Seladin-1 gene promoter by T_3_ could be TR isoform specific and found that TR-β but not TR-α is responsible for the positive regulation (Figure 4A). The gene promoter investigated in the current study contains two consensus TRE-half sites; site 1 and 2. Through the analysis of deletion mutant promoters and EMSAs, we have identified site 2 as a novel positive TRE. Estrogen receptor (ER) is another nuclear receptor closely related to Seladin-1 gene expression. Estrogens are well known to exert neurotropic and neuroprotective effects *in vitro* [55], [56] and increase Seladin-1 gene expression in fetal neuroepithelial cells [57]. Similar to site 2 for TR, half-palindromic estrogen response element (ERE) in Seladin-1 gene promoter is functionally responsible for the promoter activation by ER [58].

The ChIP assays using HTB185 cells revealed that TR-β is recruited to site 2 flanking region and that the recruitment is augmented upon T_3_ administration. Generally, TR-β as a type 2 nuclear receptor should constitutively bind to DNA in the gene promoter in the presence or absence of T_3_; however, recently, Liu et al. reported that TR-β was recruited to the TRE in a T_3_-dose-dependent manner in a time-course study [59]. Our data regarding TR-β recruitment is congruent with this report by Liu et al. The study employing several types of TR-β mutant revealed that the positive regulation of the promoter by TR-β required ligand binding, DNA binding, and heterodimerization with RXR-α, and that co-activator binding does not seem to be necessarily required for the regulation (Figure 4F).

Crosstalk between TR-β and LXR-α has been reported, especially on lipid metabolism-related genes. Recently, several types of crosstalk between TRs and LXRs have been identified and crosstalk has also been observed in other physiological systems such as central nervous system other than lipid metabolism [5], [41], [48]. Therefore, we hypothesized that LXR-α could compensate to maintain mouse Seladin-1 gene expression in hypothyroid status and in the TRKI mice. Supporting our hypothesis, TO, an artificial agonist of LXRs, induced the gene expression in a hypothyroid state (Figure 2C, 2D) and in TRKI mice (Figure 3C, 3D), but not in euthyroid state (Figure 2A, 2B). Since TO clearly increased Seladin-1 gene expression in HTB185 cells through intrinsic LXRs and induced the mouse Seladin-1 gene promoter activity in CV-1 cells, Seladin-1 is considered as an LXR target gene. Wang et al. reported that Seladin-1 gene expression in the brain of LXR-β knockout mice was not decreased compared to that of wild-type mice [34]. We speculate that it is due to that TR-β dominantly regulates Seladin-1 gene expression in mice. Another speculation is that LXR-β could not be required for the regulation of mouse Seladin-1 gene in forebrain.

**Figure 9:**
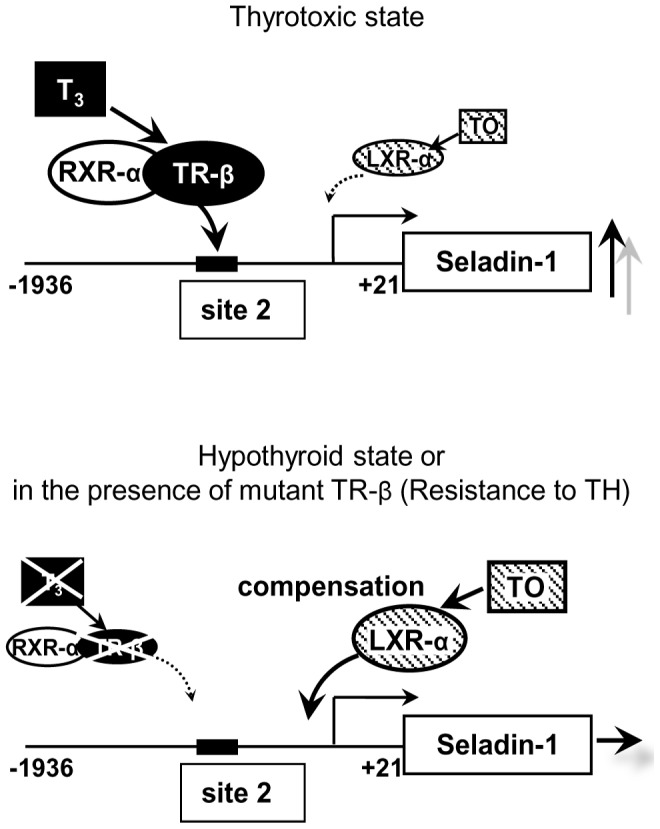
Schematic diagram illustrating the hypothetical mechanism of TR and LXR crosstalk on the mouse Seladin-1 gene promoter.

We demonstrated that LXR-α is a dominant isoform in HTB185 cells, in which Seladin-1 gene expression was positively regulated by TO (Figure 6), therefore we employed LXR-α to elucidate a molecular mechanism for the positive regulation of the gene. Based on the data obtained by the analysis of deletion constructs of the gene promoter, LXR-α activated the mouse Seladin-1 gene promoter and the responsible region should be located from −120 to −102 bp. The exact sequence of the region is GAGCATCCCGGTTCCCGC, which does not match either canonical TRE or LXRE. We performed EMSAs employing various probes with different sequence in the region; however, LXR-α or RXR-α/LXR-α heterodimer did not bind to any probes. To examine LXR-α recruitment *in vivo*, we tried to perform *in vivo* ChIP assays using mouse forebrain as we previously reported with liver [37], [41], [43], however, it did not work probably due to excessed lipid contents in the tissue. Therefore, alternatively we employed HTB185 cells to recapitulate physiological environment utilizing intrinsic nuclear receptors. Since HTB185 cells are derived from human medulloblastoma, we had to introduce the mouse Seladin-1 gene promoter as described above. The ChIP assays demonstrated that LXR-α was clearly recruited to the region from −226 to +21 bp. Even though RXR-α was also recruited to the region, it remains unclear whether LXR-α recruitment requires RXR-α in the current study. Collectively, we speculated that LXR-α is recruited to the region without DNA binding. Since site 2 is located upstream of the region from −120 to −102 bp, TR-β and LXR-α do not share the same response element in the mouse Seladin-1 gene promoter. The ChIP assays also demonstrated that LXR-α recruitment was deteriorated upon TH administration indicating that in thyrotoxic state, LXR-α could not induce the gene promoter. On the basis of these several lines of evidence, we speculate that in a thyrotoxic state, TR-β binds to site 2 and regulates Seladin-1 gene expression; on the other hand, LXR-α may functionally compensate for TR-β being recruited to the region from −120 to −102 bp under hypothyroid state or in the presence of mutant TR-β to maintain Seladin-1 gene expression (Figure 9). In hypothyroid status, co-repressors such as NCoR and SMRT [60], [61] are hard to dissociate from TR-β because of lack of T_3_. Co-repressors are tethered to TR-β Δ337T mutant, which TRKI mice harbor, because of total deficiency of ligand binding of the mutant receptor. Indeed, NCoR binds to both TR-β and LXR-α and is a key regulator of TR and LXR target genes [62]. Therefore, considering the data obtained from analysis of TRKI mice and luciferase assay using TR-β Δ337T mutant, we hypothesize that in hypothyroid state and in RTH, co-repressors should be tethered to TR-β in a squelching fashion and LXR-α are relatively free from co-repressors, which enables to activate the mouse Seladin-1 gene promoter upon administration of the LXR specific ligand.

Nowadays, the concept of subclinical hypothyroidism is widely accepted [63], [64], and it is assumed that AD patients with subclinical hypothyroidism could be increasing in number. On the basis of the data in the current study, for these patients, LXR agonists could be applicable as novel molecular therapeutic agents against AD since LXR compensates Seladin-1 gene expression under hypothyroid status.

In conclusion, mouse Seladin-1 gene expression is positively regulated by TR-β and LXR-α at the transcriptional level and LXR-α compensates the gene expression in hypothyroidism and RTH. Thus, we have, for the first time, demonstrated TR/LXR crosstalk on the Seladin-1 gene promoter in the mouse forebrain. We believe that further analysis of the crosstalk could shed light on the role of TH and LXR agonist against AD.
